# Incidence and influence factors of venous thromboembolism in traumatic rib fracture patient: a multicenter study

**DOI:** 10.1186/s13018-024-04622-1

**Published:** 2024-02-23

**Authors:** Dongsheng Zhang, Yi Yang, Yunfeng Yi, Dongbin Wang, Lei Jiang, Hai Huang, Longyu Jin, Hui Meng, Fei Xia, Guangwei Guo

**Affiliations:** 1 Department of Cardiothoracic Surgery, Shijiazhuang Third Hospital, Shijiazhuang, 050000 Hebei China; 2 Department of Thoracic Surgery, Shanghai Jiao Tong University School of Medicine Affiliated Sixth People’s Hospital, Shanghai, 200233 China; 3 Department of Cardiothoracic Surgery, Dongnan Hospital of Xiamen University, School of Medicine, Xiamen University, Zhangzhou, 363000 Fujian China; 4 Department of Cardiothoracic Surgery, Tianjin Hospital Affiliated to Tianjin University, Tianjin, 300211 China; 5 The Second Department of Surgery (Thoracic and Breast Department), Foshan Hospital of Traditional Chinese Medicine, Foshan, 528000 Guangdong China; 6 Department of Thoracic Surgery, Fuzhou Second Hospital, Fuzhou, 350007 Fujian China; 7 Department of Thoracic Surgery, The Third Xiangya Hospital of Central South University, Changsha, 410000 Hunan China; 8 Department of Thoracic Surgery, The Fifth Affiliated Hospital of Zunyi Medical University, Zhuhai, 519100 Guangdong China; 9 Department of Emergency Surgery, The Affiliated Hospital of Guizhou Medical University, Guiyang, 550001 Guizhou China; 10 Department of Cardiothoracic Surgery, Second Hospital of Shanxi Medical University, Taiyuan, 030000 Shanxi China

**Keywords:** VTE, Rib fracture, Influencing factors, Epidemiological study

## Abstract

**Background:**

This study aimed to determine the incidence and influencing factors of venous thromboembolism (VTE) in patients with traumatic rib fractures.

**Methods:**

The retrospective study analyzed medical records of patients with traumatic rib fractures from 33 hospitals.

**Results:**

The overall incidence of VTE in hospitalized patients with traumatic rib fractures was 8.1%. Patients with isolated traumatic rib fractures had a significantly lower incidence of VTE (4.4%) compared to patients with rib fractures combined with other injuries (12.0%). Multivariate analysis identified the number of rib fractures as an independent risk factor for thrombosis. Surgical stabilization of isolated rib fractures involving three or more ribs was associated with a lower VTE incidence compared to conservative treatment.

**Conclusions:**

Patients with rib fractures have a higher incidence of VTE, positively correlated with the number of rib fractures. However, the occurrence of thrombosis is relatively low in isolated rib fractures. Targeted thromboprophylaxis strategies should be implemented for these patients, and surgical stabilization of rib fractures may be beneficial in reducing the risk of VTE.

## Background

Venous thromboembolism (VTE) refers to a condition characterized by abnormal coagulation of blood within the veins, leading to impaired venous flow. VTE represents a potentially life-threatening complication following trauma, including both types of pulmonary embolism (PE) and deep vein thrombosis (DVT) [1]. In 1856, Virchow proposed three triggering factors for VTE formation: hypercoagulability of blood, stasis of venous flow, and endothelial injury [2]. Patients with traumatic rib fractures are prone to VTE occurrence due to the impact of trauma, limited mobility, and various treatment interventions. These factors can lead to venous injury, reduced blood flow, and a hypercoagulable state, thereby increasing the risk of VTE.

Reported incidence rates of trauma-related VTE range from 1 to 58%, depending on patient populations and demographic characteristics, leading to increased mortality and complications [3]. Chest trauma accounts for over 25% of all injuries, with rib fractures comprising 50–70% of chest trauma cases [4]. Studies have shown significant variation in the occurrence of DVT based on the site of fracture [5]. However, there is currently limited reported data on the actual incidence of VTE and related risk factors specifically in patients with acute chest trauma, particularly rib fractures. Despite the existence of numerous consensus statements and guidelines on VTE prevention and treatment, there is still a lack of evidence-based treatment strategies specifically tailored for patients with rib fractures. Early clinical symptoms of VTE are often subtle, making timely diagnosis challenging. However, partial thrombus organization occurs within a short period, emphasizing the importance of early diagnosis [6]. Investigating the incidence of VTE and associated risk factors in patients with traumatic rib fractures can effectively aid in the early clinical diagnosis and improve diagnostic efficiency for this condition.

Therefore, this study conducted a retrospective analysis of hospitalized patients with traumatic rib fractures in 33 hospitals affiliated with the China Chest Injury Research Society (CCIRS) between October 2020 to September 2021, aiming to determine the incidence of VTE in patients with traumatic rib fractures and further investigate the factors contributing to VTE formation in this population, so as to provide valuable insights for the development of targeted VTE prevention and management measures in patients with traumatic rib fractures.

## Study design

### Study population

This retrospective study analyzed the medical records of patients with traumatic rib fractures who were admitted and treated in the departments of participating members of CCIRS [7] from October 2020 to September 2021. The participating hospitals were located primarily in provincial capitals or other key cities in China and were either provincial key tertiary referral centers or urban key trauma treatment hospitals. Traumatic rib fractures, as defined in this study, referred to rib fractures caused by blunt or penetrating trauma, such as motor vehicle accidents, falls, sports-related injuries, assaults, or occupational accidents. The inclusion criteria encompassed patients with confirmed diagnoses of traumatic rib fractures based on medical records, radiographic imaging, or clinical documentation. However, patients with a prior history of VTE, known hypercoagulable disorders, current anticoagulant therapy, active malignancy, or contraindications to anticoagulation were excluded. Additionally, patients with severe traumatic injuries requiring immediate surgical intervention were not included. Non-traumatic rib fractures resulting from underlying medical conditions, incomplete or insufficient medical records, iatrogenic rib fractures, and severe chest wall deformities or congenital rib abnormalities were also excluded. Ethical approval for this study was obtained. The study design involved a retrospective analysis of existing medical records and did not involve direct patient contact or intervention. All patient information was handled confidentially, and personal identifiers were removed to ensure privacy and compliance with relevant regulations.

### Data collection

Each participating member reviewed the medical records of patients with traumatic rib fractures admitted to their respective departments for baseline entry. Baseline clinical parameters of the enrolled patients were collected and summarized, amounting to a total of 34 items. These parameters included: (1) patient demographic data, such as age, gender, body mass index (BMI), and smoking status; (2) disease-related data, including diabetes mellitus, hypertension, prophylactic drug anticoagulation, number of rib fracture roots, presence of combined cranial injury, combined vertebral fracture, combined pelvic fracture, combined abdominal organ injury, upper limb fracture, lower limb fracture, and whether surgical stabilization of rib fractures was performed; and (3) laboratory test results, including D-dimer, prothrombin time (PT), activated partial thromboplastin time (APTT), plasma fibrinogen (Fbg), hemoglobin, platelet count at 24, 48, 72 h, as well as other relevant indicators. Additionally, the prophylactic pharmacological anticoagulation protocol consisted of administering low-molecular heparin at a dosage of 100 units per kilogram of body weight, once a day. This regimen was continued until the patient could walk normally on the ground or until a new thrombus was detected. It was important to note that the choice of fixation method was determined by the practices of each participating center, resulting in a variation of surgical techniques [rib plate screw fixation or rib ring hugger (rib claw)] across the study cohort.

### Diagnostic criteria of VTE

While lower extremity venography is considered the gold standard for diagnosing DVT, it is not routinely used in clinical practice due to its invasive nature. In this study, all included patients underwent routine lower limb venous color Doppler ultrasound on the day of admission. Follow-up examinations or venous angiography were performed if significant symptoms or special requirements arose. The diagnosis of DVT was established through color Doppler ultrasound or angiography. Similarly, all cases of PE were diagnosed and confirmed using pulmonary vascular enhancement CT scans.

### Statistical analysis

Statistical analysis was conducted using SPSS 24.0 software. Quantitative indicators were presented as mean ± standard deviation (SD). The independent sample t test or independent sample rank sum test was utilized for comparisons between two groups, while the analysis of variance or Kruskal–Wallis H rank sum test was employed for comparisons between multiple groups. Qualitative indicators were described using frequencies (composition ratio), and the chi-square test (*χ*^2^ test) was used for comparisons between groups. Multifactor analysis was performed using binary logistic regression analysis, with inclusion criteria of 0.05 and exclusion criteria of 0.1. All hypothesis tests were two-sided with a significant level (*α*) of 0.05. Differences were considered statistically significant at *P* < 0.05.

## Results

### Baseline characteristics

The study included case data from hospitals affiliated with CCIRS members, encompassing a total of 5774 cases of traumatic rib fractures across 33 hospitals in 14 provinces (Liaoning, Hebei, Shanxi, Shandong, Shaanxi, Hunan, Sichuan, Guangdong, Zhejiang, Jiangsu, Fujian, Yunnan, Qinghai, and Guizhou), one autonomous region (Inner Mongolia Autonomous Region), and four municipalities (Beijing, Shanghai, Tianjin, and Chongqing) in China. Among them, 466 patients experienced in-hospital VTE, resulting in an overall VTE incidence of 8.1%. There were 11 cases of in-hospital PE, of which six cases were combined with DVT and five cases were not. The incidence of PE was 0.19%. The total number of deaths was 11, with causes of death including craniocerebral injury, thoracoabdominal organ injury, and hemorrhagic shock, among others. There were no deaths directly attributed to thrombosis-related complications. All patients with rib fractures were divided into two groups: the control group (without in-hospital VTE) and the case group (with in-hospital VTE). A detailed comparison of baseline characteristics between these groups is presented in Table 1.

**Table 1:** Baseline characteristics

Variables	Group		*U*/*x*^2^	*P*
	Control (n = 5308)	VTE (n = 466)		
Age	55.1 ± 13.7	59.3 ± 13.3	1,026,835.000	< 0.001
Female	1659 (31.3)	150 (32.2)	0.174	0.667
BMI	23.6 ± 3.0	24.0 ± 2.9	1,145,293.000	0.008
Smoking history	1538 (29.0)	163 (35.0)	7.430	0.006
Diabetes	497 (9.4)	60 (12.9)	6.063	0.014
Hypertension	943 (17.8)	99 (21.2)	3.506	0.061
Prophylactic drug anticoagulation	1309 (24.7)	399 (85.6)	764.273	< 0.001
Cranial trauma	515 (9.7)	93 (20.0)	47.817	< 0.001
Vertebral fractures	853 (16.1)	134 (28.8)	48.642	< 0.001
Pelvic fractures	451 (8.5)	83 (17.8)	44.284	< 0.001
Abdominal organ injuries	361 (6.8)	50 (10.7)	10.000	0.002
Lower extremity fractures	491 (9.3)	156 (33.5)	252.695	< 0.001
Upper extremity fractures	1051 (19.8)	115 (24.7)	6.325	0.012
Internal rib fixation	1906 (35.9)	204 (43.8)	11.438	0.001
Ventilator-assisted ventilation	158 (3.0)	72 (15.5)	174.283	< 0.001
*D-dimer*				
24 h	2.3 ± 7.0	5.7 ± 10.1	894,028.500	< 0.001
48 h	0.9 ± 29.4	1.1 ± 3.5	1,118,441.000	< 0.001
72 h	0.3 ± 1.5	1.0 ± 3.3	1,152,414.500	< 0.001
*PT*				
24 h	9 ± 6.2	10.7 ± 6.9	1,056,246.500	< 0.001
48 h	2.2 ± 4.8	3.2 ± 6.6	1,129,824.000	< 0.001
72 h	1.4 ± 3.9	2.4 ± 5.9	1,132,958.000	< 0.001
*APTT*				
24 h	21.7 ± 14.2	23.5 ± 12.5	1,199,991.000	0.282
48 h	5.0 ± 11.2	7.0 ± 13.1	1,142,262.000	< 0.001
72 h	3.2 ± 9.2	5.7 ± 12.0	1,124,108.500	< 0.001
*Fbg*				
24 h	2.4 ± 4.7	2.8 ± 4.6	1,232,428.500	0.899
48 h	0.6 ± 1.4	1.0 ± 2.4	1,129,173.000	< 0.001
72 h	0.5 ± 1.6	0.8 ± 1.9	1,139,148.000	< 0.001
*Hemoglobin*				
24 h	92.2 ± 64.0	93.1 ± 51.7	1,071,106.500	< 0.001
48 h	30.3 ± 54.0	29.0 ± 47.3	1,228,558.000	0.756
72 h	26.3 ± 50.4	28.1 ± 46.1	1,195,656.000	0.111
*Platelets*				
24 h	156.4 ± 109.5	165.6 ± 102.7	1,179,955.000	0.097
48 h	51.0 ± 90.2	50.9 ± 89.8	1,218,778.000	0.510
72 h	41.4 ± 86.2	52.4 ± 92.8	1,143,025.000	< 0.001

### Comparison of VTE incidence between the isolated rib fractures group and the rib fractures combined injury group

The patients with rib fractures were further divided into the isolated rib fractures group and the rib fractures combined injury group included cases where rib fractures were accompanied by craniocerebral injury, abdominal organ injury, upper extremity fracture, lower extremity fracture, vertebral fracture, or pelvic fracture. As shown in Table 2, among the 2964 patients with rib fractures from chest trauma alone, 130 (4.4%) experienced VTE, while among 2810 patients with rib fractures combined with other trauma, 336 (12.0%) experienced VTE. The incidence of VTE was significantly lower in the isolated rib fracture group compared to the rib fracture combined injury group (4.4% vs. 12.0%, *P* < 0.01).

**Table 2:** Comparison of VTE rates between the isolated rib fractures group and the rib fractures combined injury group

Group	Thrombosis	No thrombosis	Total	*x*^2^	*P*
Isolated rib fractures	130 (4.4%)	2834 (95.6%)	2964	111.452	< 0.001
Rib fracture combined injury	336 (12.0%)	2474 (88.0%)	2810		
Total	466 (8.1%)	5308 (91.9%)	5774		

### Multifactorial analysis of the occurrence of VTE in all cases

A binary logistic regression model was used to perform multifactor analysis on a total of 17 factors, including gender, age, BMI, diabetes mellitus, hypertension, smoking, prophylactic drug anticoagulation therapy, number of rib fracture roots, combined craniocerebral trauma, combined vertebral fracture, combined pelvic fracture, combined abdominal organ injury, upper extremity fracture, lower extremity fracture, whether surgical stabilization of rib fractures was performed, and whether another site surgery was performed. Table 3 presents the results of the analysis, showing that age, smoking, prophylactic drug anticoagulation therapy, number of rib fractures, combined vertebral fractures, combined pelvic fractures, combined lower extremity fractures, and ventilator-assisted ventilation were independent risk factors for VTE in patients with traumatic rib fractures. Patients who underwent internal fixation of rib fractures had a lower risk of VTE (OR = 0.219, 95% CI: 0.164–0.292, *P* < 0.001), suggesting that internal rib fixation surgery is a protective factor against thrombosis.

**Table 3:** Multifactorial analysis of the occurrence of VTE in all cases

Risk factors	*β*	SE	Wald *x*^2^	*P*	OR	95% CI	
						Lower	Upper
Age	0.019	0.004	20.265	< 0.001	1.019	1.011	1.027
Smoking	0.359	0.120	8.864	0.003	1.431	1.130	1.813
Anticoagulation	1.002	0.113	78.112	< 0.001	2.724	2.181	3.402
Surgical stabilization of rib fractures	− 1.519	0.147	106.765	< 0.001	0.219	0.164	0.292
Number of rib fracture roots	0.787	0.090	75.862	< 0.001	2.196	1.840	2.622
Vertebral fractures	0.410	0.129	10.139	0.001	1.506	1.171	1.938
Pelvic fractures	0.499	0.165	9.106	0.003	1.647	1.191	2.278
Lower extremity fractures	1.320	0.135	94.881	< 0.001	3.742	2.870	4.881
Ventilator-assisted ventilation	1.426	0.187	57.909	< 0.001	4.161	2.882	6.007
Constant	− 5.835	0.337	300.241	< 0.001	0.003		

Logistic formula: logitP =  − 5.835 + 0.019 age + 0.359 smoking history + 1.002 anticoagulation − 1.519 surgical stabilization of rib fractures + 0.787 number of rib fracture roots + 0.410 vertebral fractures + 0.499 pelvic fractures + 1.320 lower extremity fractures + 1.426 ventilator-assisted ventilation

### Multifactorial analysis of VTE in cases of isolated rib fractures

After excluding multiple trauma-related factors, we conducted an analysis on patients with isolated rib fractures using a similar approach to the methods mentioned above. Table 4 presents the results, indicating that age, history of smoking, prophylactic drug anticoagulation therapy, number of rib fracture roots, and ventilator-assisted ventilation remained independent risk factors for thrombosis in patients with isolated rib fractures. Internal fixation of rib fractures reduced the incidence of VTE (OR = 0.199, 95% CI: 0.118–0.335, *P* < 0.001).

**Table 4:** Multifactorial analysis of VTE in cases of isolated rib fractures

Risk factors	*β*	SE	Wald *x*^2^	*P*	OR	95% CI	
						Lower	Upper
Age	0.019	0.007	6.936	0.008	1.019	1.005	1.034
Smoking	0.538	0.204	6.972	0.008	1.713	1.149	2.555
Anticoagulation	1.386	0.199	48.665	< 0.001	4.001	2.710	5.906
Surgical stabilization of rib fractures	− 1.614	0.265	37.111	< 0.001	0.199	0.118	0.335
Number of rib fracture roots	0.950	0.167	32.342	< 0.001	2.587	1.864	3.589
Ventilator-assisted ventilation	2.561	0.464	30.509	< 0.001	12.946	5.218	32.120
Constant	− 6.600	0.586	126.900	< 0.001	0.001		

Logistic equation: logitP =  − 6.600 + 0.019 age + 0.538 history of smoking + 1.386 anticoagulation − 1.614 surgical stabilization of rib fractures + 0.9507 number of rib fracture roots + 2.561 ventilator-assisted ventilation

### Effect of internal fixation surgery for rib fractures on the incidence of thrombosis

Patients with isolated rib fractures were further divided into three groups based on the number of rib fractures: 1–2 rib fractures, 3–6 rib fractures, and ≥ 7 rib fractures. The incidence of thrombosis was compared between surgical treatment with internal fixation of rib fractures and conservative treatment in each group. As shown in Table 5, there was no statistical difference in the incidence of thrombosis between surgical and conservative treatment in the 1–2 rib fracture group (0 vs. 1.5%, *P* = 0.786). In the 3–6 and ≥ 7 rib fracture subgroups, surgical treatment with internal fixation showed a significantly lower incidence of thrombosis compared with conservative treatment (1.8% vs. 5.5%, 3.8% vs. 12.1%, *P* < 0.001).

**Table 5:** Effect of internal fixation of rib fractures on the incidence of VTE

Number of rib fractures roots	Surgical	Conservative	*x*^2^	*P*
1–2	0 (0.0%)	7 (1.5%)	0.087	0.786
3–6	13 (1.8%)	59 (5.5%)	14.860	< 0.001
≥ 7	13 (3.8%)	38 (12.1%)	16.076	< 0.001

## Discussion

After traumatic fractures, imbalances in the coagulation system can promote a hypercoagulable state of blood, manifested by vascular endothelial damage, excessive thrombin synthesis, hyperfibrinogenemia, platelet hyperactivity, impaired anticoagulation system, and shutdown of the fibrinolytic system [8, 9]. Bed rest and limited mobility following fractures can lead to venous blood flow stasis, resulting in tissue edema, which in turn affects venous return. Venous injury, venous stasis, and hypercoagulable blood are considered the three elements contributing to venous thrombosis formation [10]. Therefore, fractures themselves can be considered as the initiating factor of DVT [11]. The incidence of VTE is higher in patients with fractures [12, 13], and the occurrence rates of VTE differ significantly depending on the fracture location. Sun Ning et al. [14] reported the following descending order of DVT occurrence rates based on fracture sites: multiple fractures (29.6%) > pelvic and acetabular fractures (21.1%) > proximal and middle femur fractures (20.0%) > periarticular knee fractures (17.8%) > lower leg fractures (10.3%) > ankle fractures (2.2%). In addition, multiple rib fractures can also cause severe pain and destabilization of the chest wall. Insufficient expansion of the chest due to chest belt fixation leads to difficulty in sputum clearance, resulting in atelectasis and pneumonia, thereby increasing the risk of VTE [15]. Therefore, traumatic rib fractures can lead to VTE occurrence. Our research findings showed an isolated rib fracture VTE occurrence rate of 4.4%, which is consistent with the aforementioned conclusions. Furthermore, compared to rib fractures combined with other injuries (12.0%) and fractures in the pelvis, spine, and femur, rib fractures exhibit a relatively lower incidence of VTE. This may be attributed to their limited trauma confined to the chest, with fewer associated injuries to the veins of the limbs, particularly the lower limb veins that are prone to thrombosis. Additionally, chest trauma does not require measures such as tourniquets, restraint, or prolonged bed rest. The mild venous stasis allows for early recovery exercises, promoting blood circulation and the restoration of the normal flow status, thus reducing the occurrence of VTE.

Our study also found that PE events associated with rib fractures did not seem to be entirely coupled with the occurrence of DVT. Among the 11 patients who experienced PE events in this study, DVT was not detected in five cases. It has been indicated that post-traumatic DVT and PE may represent a broad pathological process of thrombus formation [16]. Local inflammation, occult vascular injury, low flow state, depletion of protein C, hypercoagulable state, and adrenergic responses after chest trauma can lead to endothelial inflammation and the generation of circulating adhesion molecules, resulting in local thrombosis and rapid occlusion, which may be a contributing factor to the “in situ” formation of PE [6, 17]. An analysis of 2370 trauma patients showed that among the 30 cases of PE, 19 occurred without DVT [18]. Modeling PE as the primary event showed youthfulness, non-severe injuries (injury severity score < 15), central venous catheter placement, and prophylactic heparin, compared to the model focusing on VTE as the primary risk, indicating that these events are distinct in clinical terms. This could be attributed to the unique mechanism of new thrombus formation following trauma leading to pulmonary arterial thrombosis [19]. The inflammatory process triggered by chest trauma forms the basis for the occurrence of PE, while tissue hypoxia resulting from lung injury may exacerbate this process. This may explain why chest trauma is associated with a higher incidence of PE compared to other injuries but not necessarily a higher incidence of DVT [6].

Previous studies have already demonstrated a close association between patient age, smoking history, multiple fractures, and mechanical ventilation support with the occurrence of VTE [20, 21], and these risk factors also apply to patients with rib fractures. Additionally, this study also revealed that the number of rib fractures and the choice between surgical or conservative treatment for rib fractures also influenced the occurrence of thrombosis. Patients with a higher number of rib fractures exhibited a higher incidence of VTE, further confirming the consensus that the more severe the trauma, the higher the incidence of VTE. An analysis of 64,750 rib fracture patients from the 2005 National Trauma Data Bank (NTDB) in the USA [22] showed that with each additional rib fracture, the incidence and mortality of pneumonia, ARDS, pneumothorax, resorptive pneumonia, and empyema increased. Complications such as pneumonia and ARDS are also associated with the occurrence of VTE, which may explain why the number of rib fractures affects the occurrence of VTE.

Surgical stabilization of rib fractures has been shown to alleviate chest pain in patients [4], as well as help to shorten the duration of mechanical ventilation, reduce the risk of sepsis, shorten hospital stays and intensive care unit stays, and decrease the incidence of pneumonia [23]. Along with mitigating the adverse stress responses associated with pain reduction, surgical stabilization also facilitates a reduction in bed rest, earlier ground activity, and pulmonary function exercise, thus contributing to a decreased occurrence of VTE. This positive impact of surgical stabilization on VTE prevention provides a basis for considering more proactive strategies for rib fracture fixation in the future.

Research has demonstrated that the incidence of DVT is significantly lower in trauma patients receiving pharmacological prophylaxis compared to those who do not receive such preventive measures [24]. The American College of Chest Physicians and the Eastern Association for the Surgery of Trauma recommend the use of low-molecular-weight heparin for DVT prophylaxis in trauma patients [25, 26]. Our study findings indicate that prophylactic anticoagulation with medication is a risk factor for VTE in patients with rib fractures. This may be attributed to the clinical practice of physicians who are more inclined to administer prophylactic anticoagulation treatment to patients with higher VTE risk factors, such as severe trauma, advanced age, and a history of prior embolic events. Furthermore, doctors tend to be more vigilant in monitoring VTE occurrences in patients who have been selected for prophylactic anticoagulation therapy compared to those who have not received such treatment, which is directly associated with more frequent screening for DVT using venous color Doppler ultrasonography, potentially resulting in a higher diagnostic rate of VTE.

The use of anticoagulation therapy not only increases healthcare costs, but also poses a risk of intraoperative bleeding. As a result, there is still controversy regarding the routine administration of anticoagulation therapy for fracture patients, both domestically and internationally. In North America, uncomplicated isolated fractures do not typically warrant routine pharmacological DVT prophylaxis [27, 28]. However, in Europe, as well as in China, routine VTE prophylaxis is more common [18, 29, 30]. It is worth noting that the guidelines of the American Association for Thoracic Surgery discourage routine anticoagulation therapy for patients with uncomplicated isolated fractures [18, 29, 30], whereas the British guidelines recommend its routine use [31]. The Chinese Guidelines for Prevention of VTE in Major Orthopedic Surgery emphasize that due to the high risk of VTE occurrence in postoperative orthopedic patients, the thrombotic and bleeding risks should be fully weighed, and anticoagulation therapy should be administered rationally [32]. Currently, there is a lack of specific guidelines addressing the need for routine anticoagulation therapy in patients with rib fractures. Considering the relatively low incidence of VTE observed in this study involving isolated rib fractures, we recommend a cautious approach to the selection of prophylactic anticoagulation therapy, weighing the pros and cons, and taking into account other VTE risk factors that may be present in each individual patient. Overall, in clinical treatment, considering the characteristics of VTE occurrence in thoracic trauma, both the attention degree and the preventive and treatment strategies should be approached differently compared to traumatic orthopedics.

As a retrospective study, this study also has certain limitations. Firstly, different hospitals have varying admission criteria for patients with rib fractures. Although the number of rib fractures was recorded, it did not capture the severity of fracture displacement, as well as the presence of associated conditions such as pneumothorax, hemothorax, or vascular organ injuries. Secondly, the choice between conservative and surgical treatment for rib fractures is influenced by patient-specific factors, including personal experiences, family economic situation, and knowledge of surgery. This introduces potential bias in the selection of subjects. Thirdly, there are variations among different centers regarding the selection of surgical indications for rib fractures and the protocols for VTE prevention (timing, choice of medications, patient education, and physical therapy methods), making it difficult to standardize and control intervention factors. Fourthly, the timing of lower extremity venous ultrasound and pulmonary artery-enhanced CT scans may differ, inevitably leading to certain cases of missed diagnosis and introducing observation bias. Considering these characteristics of retrospective studies, our research is subject to limitations such as sample selection bias, differences in baseline data among samples, lack of uniform intervention factors, and outcome variable standards, which may introduce confounding factors and biases in the study results. Finally, the absence of a detailed breakdown of the number of cases and the percentage distribution of specific surgical techniques, such as rib plate screw fixation and rib ring hugger (rib claw) resulting in a lack of granularity in our analysis, may limit a comprehensive understanding of how variations in surgical methods could potentially impact the final outcomes. Future studies should aim to address this limitation by incorporating a more detailed exploration of the specific surgical techniques employed and their potential influence on treatment outcomes. Even so, this study provides valuable information on the epidemiology of VTE in patients with traumatic rib fractures, which can contribute to the rational application of public health and clinical resources for the prevention, diagnosis, and treatment of VTE in thoracic trauma. Future multicenter prospective studies are necessary to gather more evidence on this topic.

## Conclusion

Patients with rib fractures have a higher incidence of VTE, which is positively correlated with the number of rib fractures. However, the occurrence of thrombosis is relatively low in patients with isolated rib fractures without other associated injuries, suggesting that a more targeted approach to thrombosis prevention should be considered for patients with isolated rib fractures, rather than simply adopting the current preventive measures used in orthopedic trauma. Surgical stabilization of rib fractures may be beneficial in reducing the risk of VTE.

## Data Availability

All data are available from the corresponding author on reasonable request.

## Abbreviations

VTE: Venous thromboembolism
PE: Pulmonary embolism
DVT: Deep vein thrombosis
CCIRS: China Chest Injury Research Society
BMI: Body mass index
PT: Prothrombin time
APTT: Activated partial thromboplastin time
Fbg: Plasma fibrinogen
